# Cost Considerations for Rapid Start Implementation: Analysis from Seven Rapid Start Provider Sites

**DOI:** 10.1007/s10461-026-05069-7

**Published:** 2026-04-28

**Authors:** Pamela M. Murnane, Starley B. Shade, Monika Damle, Thomas Donohoe, Dawn Middleton, Sara Friedman, Tony Jimenez, Kendall Brooks, Lindsay Senter

**Affiliations:** 1 Department of Epidemiology & Biostatistics, University of California, San Francisco, San Francisco, CA USA; 2 Institute for Global Health Sciences, University of California, San Francisco, San Francisco, CA USA; 3 Cicatelli Associates, Inc, New York, NY USA; 4 Department of Family Medicine, David Geffen School of Medicine at UCLA, 550 16th Street, Los Angeles, CA 94158 USA

**Keywords:** HIV infections, Treatment initiation, Costs and cost analysis

## Abstract

Antiretroviral treatment (ART) initiation within days of HIV diagnosis, also known as “Rapid Start”, improves linkage to care, virologic suppression and retention in care. However, data on the costs of Rapid Start implementation in the United States are limited. We aimed to estimate the costs and cost-effectiveness of Rapid Start among seven geographically diverse Ryan White-funded programs with broad variability in populations served and services offered. Each site estimated the costs of ART initiation services, management, and Rapid Start planning activities in three 12-month periods: pre-, initial, and sustained implementation. We estimated quality-adjusted life years (QALY) saved, incremental costs per-person and incremental cost per QALY saved during implementation. We considered an intervention cost-effective if it cost <$76,399 USD per QALY saved (US per capita GDP, 2021). The number of persons initiating ART increased at most sites during implementation, which, together with assumed shorter times to viral suppression contributed to QALYs saved. Pre-implementation, the per-person cost of ART initiation and Rapid Start planning ranged from $549-$30,626, which declined at most sites during implementation. Incremental costs per-person ranged from a cost savings of -$21,284 up to $23,850 additional costs during the initial year and from $24,303 savings up to $11,774 additional costs during sustained implementation. The cost-effectiveness threshold was met by five sites during initial implementation and by all seven during sustained implementation. In conclusion, we demonstrated that Rapid Start was cost effective across a wide range of clinical settings, further bolstering this model’s importance in ending the HIV epidemic.

## Introduction

 Antiretroviral treatment (ART) for persons living with HIV is essential for lifelong health and prevention of onward transmission. Early ART initiation, prior to significant disease progression, further optimizes health [1–3] and prevention benefits [4] and is now recommended globally [5, 6]. Importantly, the Ending the HIV Epidemic Initiative in the United States prioritizes early ART initiation as a central strategy towards reducing new HIV infections by 90% by 2030 [7]. 

From 2013 to 2017, San Francisco Department of Public Health implemented “Rapid” ART initiation, defined as initiation within seven days of diagnosis and ideally on the same day [8]. Implementation of Rapid ART initiation requires adaptations to traditional HIV care models to reduce the time between diagnosis and treatment initiation, including modified counseling and testing, increased communication between HIV prevention and treatment programs, patient navigation support, and minimization of pre-treatment laboratory testing and appointments. This first in the country program demonstrated improved linkage to care, averting the risk of care disengagement between the time of diagnosis and ART start [9]. Now known as “Rapid Start”, this model has been implemented in many settings across the United States which have demonstrated shorter times to virologic suppression, [8, 10–12] improved retention in care, and sustained virologic suppression up to 12 months following ART initiation [13, 14]. 

Although the clinical and public health benefits of Rapid Start are well established, few studies have examined the costs of implementation. In a similar early ART start model, one study in South Africa found that initiation on the same day that clinical eligibility criteria were met (e.g., CD4 thresholds) cost an additional $18 USD per virally suppressed patient compared to later start [15]. More recently, a study in China estimated cost savings over 20 years of follow-up after Rapid ART (within 7 days) compared to later start in a Markov model [16]. In the United States, a study of Medicaid-covered patients found that those who initiated ART within 14 days of diagnosis had lower per-patient per-month overall health care costs (not just HIV care) over the subsequent three years compared to those who initiated more than 6 months following diagnosis ($651 vs. $1,196) [17]. However to date, no studies have estimated the implementation costs and cost-effectiveness of Rapid Start programs in the United States, where Rapid Start also requires adaptations to services related to health insurance benefits and payments given the unique insurance framework in this setting.

To address this gap, we worked with seven Ryan White HIV/AIDS Program (RWHAP)-funded sites to estimate the additional (or incremental) costs associated with planning, service provision, and management of Rapid Start to quantify the potential cost-effectiveness of these programs. This work was nested within our recently completed comprehensive environmental scan of existing Rapid Start programs aiming to identify, disseminate, and promote replication of best practices that facilitate successful Rapid Start implementation [18]. 

## Methods

This costing study is part of the Rapid Antiretroviral Therapy Dissemination Assistance Provider Initiative, which was led by Cicatelli Associates Inc. (CAI), a non-profit global capacity building organization, in collaboration with researchers at the University of California (Los Angeles and San Francisco), and funded by Health Resources and Services Administration (HRSA) Special Projects of National Significance. From 2020 to 2021, CAI conducted a comprehensive environmental scan [19] of existing Rapid Start programs in the United States to identify best practices, which have been reported [18]. This costing study was conducted from January through June 2022 among a sub-set of sites identified in the environmental scan.

### Site Selection

To identify sites offering rapid access to HIV care and treatment for the environmental scan, CAI searched PubMed, Google Scholar, AIDS Education and Training Centers (AETCs) and sessions from the 2020 National Ryan White Conference on HIV Care and Treatment and solicited recommendations from experts in the field. We identified 130 programs and reviewed publicly available data to determine which had current functioning Rapid Start services that explicitly aimed to prescribe ART within seven days of diagnosis. Among the 65 that met these criteria, we reached 45 for phone interviews regarding Rapid Start processes, implementation facilitators and barriers, and outcomes. Based on these interview data, the team selected 18 for investigation of best practices. Inclusion criteria were: provision of Rapid Start services for at least 2 years, demonstrated improvements in linkage to care, viral suppression, and retention in care following implementation, and willingness to participate in a virtual site visit (i.e., interviews with multiple staff members). Among eligible sites, we aimed to include adequate representation of region (South, Midwest, West, or Northeast), rurality, care setting (e.g. hospital, public health clinic) implementation level (jurisdiction-based or individual clinic), Medicaid expansion state status, key program features (e.g. onsite lab, onsite pharmacy, availability of ART starter packs), and populations served (size and demographics). Finally, among the 18 sites included in the environmental scan, given limited resources, we selected eight for in-depth descriptions of their care models in the Compendium of Best Practices [18]. To select the eight, we again sought to achieve broad representation across site characteristics detailed above.

The eight sites selected for in-depth descriptions in the environmental scan were also invited to participate in the costing study. Ultimately, seven sites contributed costing data and are included in this analysis (Table 1): (1) Asian Health Services, Oakland, CA; (2) Borinquen Health Care Center, Miami, FL; (3) LGBT Life Center & CAN Community Health, Norfolk, VA; (4) Hennepin Healthcare Positive Care Center, Minneapolis, MN; (5) Kern County Health Officer’s Clinic, Bakersfield, CA; (6) The Max Clinic, Seattle, WA; and (7) University of Alabama – Birmingham 1917 Clinic, Birmingham, AL. Data regarding Rapid Start program features were collected as part of the development of the Compendium of Best Practices and are included in Table 1 for context.

**Table 1:** Site level characteristics of Ryan white HIV/AIDS programs providing rapid start ART initiation services

Site	Initial year*	Region	Rurality	Medicaid expansion state status	*N* patients in care & program level	Priority populations served	Health care setting	Rapid start program features
Asian Health Services Oakland, CA	2016	West	Urban	Yes	< 100 Single site only	Low-income and immigrant	Community Health Center/FQHC • Provides medical, dental, and mental health services for all ages in English and 14 other languages • HIV clinic provides comprehensive HIV treatment and prevention services	Strong community partnerships with clinics in the area. Warm handoffs to bring clients into clinic.
Borinquen Health Care Center in Miami-Dade county Miami, FL	2016	South	Urban	No	500-1,000 County-wide	Hispanic, Latinx, Haitian, unhoused, uninsured	Health Department/Hospital-based/FQHC Strong partnership between the Miami-Dade County RWHAP Part A and the Florida Department of Health RWHAP Part B to provide ART medication.	“One stop shop” clinic for the county. Provide testing, medical treatment, referrals for support services, and medical care.
LGBT Life Center & CAN Community Health Norfolk, VA	2019	South	Rural	Yes	500-1,000 Single site only	LGBTQ+ populations	Community Health Center Community-based organization partnership with private, not-for-profit organization to provide comprehensive HIV care	Part of state initiative to adopt Rapid Start as standard of care. Leveraged telehealth.
Positive Care Center, Hennepin Healthcare Minneapolis, MN	2018	Midwest	Urban	Yes	2,000–2,500 Single site only	Unhoused populations	Hospital-Based Clinic • HIV primary care clinic within a multi-tiered health care system Comprehensive health and psychosocial services to people with HIV.	Pharmacists are embedded within their core staffing model. Patients establish a relationship in the first visit.
Kern County Health Officers Clinic Bakersfield, CA	2019	West	Rural & suburban	Yes	< 100 County-wide	Low-income, unhoused	Health Department • Offer STI testing, PrEP/PEP services	Partner with emergency departments who link patients. Links back to community clinics for ongoing HIV care.
The Max Clinic Seattle, WA	2015	West	Urban	Yes	< 100 Single site only	Unhoused; complex medical and social needs; re-engaging in care	Community Health Center • Established in partnership with the Madison Clinic (HIV specialty treatment center), and the Public Health Sexual Health Clinic (walk-in STI clinic with comprehensive sexual health services)	Low barrier and street-based services. Substance use treatment services. Facilitate engagement with monetary incentives
University of Alabama (UAB) at Birmingham 1917 Clinic Birmingham, AL	2018	South	Urban	No	3,500–4,000 Single site only	Low-income, uninsured, under-insured	Community Health Center • Provides comprehensive HIV care • Part of a school of medicine • Large established Ryan White clinic	Onsite pharmacy provides same-day ART

* Initial year of rapid start implementation

*FQHC* federally qualified health center

### Data Collection Instruments

To develop the costing data collection tools, we first met with representatives from three Rapid Start sites to identify key activities and staff roles that contributed to routine and rapid initiation of ART. To assist sites with recall and estimation of the costs associated with these activities, we developed three standardized Excel-based data collection tools, one for each time period of interest: (1) *pre-implementation*: the year prior to implementation of Rapid Start; (2) *initial implementation*: the first year of implementation; and (3) *sustained implementation*: the most recent year of implementation (i.e. 2021). Each tool captured the number of people who initiated ART during the period of interest, including newly diagnosed and those returning to care, as well as actual and in-kind costs of ART initiation for each period as detailed below.

#### Personnel Costs

Each tool included worksheets to capture salary and benefits for all individuals involved in one or more of three overarching areas during the year: planning for Rapid Start, provision of ART initiation services (including initial patient follow-up through 8 weeks), and oversight and management of these activities. To improve the accuracy of information provided, the tools required estimates of the number of hours that each individual contributed to specific activities within these three overarching areas. Effort towards planning included stakeholder engagement, protocol development, data system revision, hiring and training. Effort towards initiation of ART included linkage to care activities, clinical visit(s) prior to initiation of ART, clinical visit for initiation/re-initiation of ART, assessment activities (i.e., housing and transportation needs, mental health, and substance use), benefits eligibility assessment and enrollment, referrals to support services, and follow-up HIV care (through eight weeks after initiation of ART). Effort towards management and oversight included provision or receipt of supervision, data monitoring and quality improvement activities, and program oversight (i.e., human resources, financial and program administration).

#### Other Costs

Each tool also included a worksheet to identify and estimate the costs of recurring goods and services associated with Rapid Start initiation, particularly costs associated with client engagement and retention (i.e., financial incentives, food vouchers/meals, mobile phones and service for clients, and transportation vouchers or services), as well as non-reimbursed costs associated with client visits and medication. Finally, although we developed a worksheet to estimate capital equipment and facility costs, we ultimately did not require sites to complete data collection for this category because implementation of Rapid Start does not require new facilities or equipment.

### Data Collection Procedures

Between January and June 2022, we met via zoom with programmatic and financial staff at each site to provide an overview of the costing activity and training on the use of the costing tools. Most sites submitted data within 3–6 weeks of training. We reviewed data to identify outliers and missing data and conducted follow-up calls with each site to present site-specific preliminary results and to address any questions or issues with submitted data. Note that the sites had implemented Rapid Start between 2015 and 2019, thus the recall period for pre- and initial implementation years varied somewhat by site. Furthermore, because the recall period was up to seven years for some sites, we consider these data to be “estimates” of costs.

### Analysis

The costing data described above together with the number of people who initiated ART during each period contribute to the analyses described below.

*Incremental Quality-adjusted life-years (QALYs) saved*. To estimate QALYs saved during initial and sustained implementation, we made the following assumptions: (1) viral suppression is achieved within 60 days following treatment initiation [20, 21] (2) Rapid Start reduces the time between diagnosis and treatment initiation by at least one week and up to one month; (3) a year of viral suppression, compared to a year of viremia, saves 0.334 QALYs (95% confidence interval [CI]: 0.273, 0.393) [22]. We calculated incremental QALYs saved for each site as follows:

**Figure:**
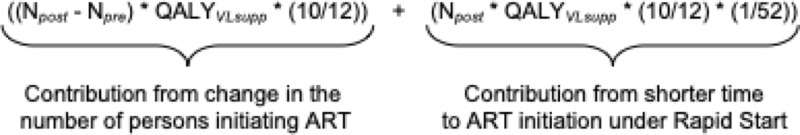

where N_*post*_ = the number of people initiating ART during Rapid Start at the site (either the initial or sustained implementation year), N_*pre*_*=* the number of people initiating ART during the pre-implementation year, QALY_*VLsupp*_ = QALYs saved for a year of viral suppression (0.334; assumption #3). The constant 10/12 represents the assumption that viral suppression is achieved within two months after ART initiation (assumption #1), and the constant 1/52 represents the assumption that Rapid Start leads to a one week reduction in time to initiation of ART (assumption #2). In a separate calculation, we replaced 1/52 with 1/12 to estimate QALYs if Rapid Start leads to a one month reduction in time to ART initiation (assumption #2), and we report both estimates.

#### Costs

For each study period, we estimated the total cost of ART initiation for each site from the data provided by sites. We then estimated the cost per-person per year (total cost divided by number of persons initiating ART), the incremental cost per-person during implementation (cost per-person in each implementation year minus cost per-person in the pre-implementation year), and incremental cost overall during implementation (total cost in each implementation year minus total cost in the pre-implementation year). We present total, per-person, and incremental costs for each site and year. Additionally, to characterize why per-person costs may differ by site during sustained implementation, we present the contribution of each type of personnel activity to the total cost.

#### Cost-Effectiveness

We estimated the incremental cost-effectiveness ratio (ICER) as the incremental cost (overall) divided by incremental QALYs saved for each implementation period and for each site. Because our calculation of QALYs could be negative if the number initiating ART declined during Rapid START, in this case we only used QALYs saved due to shorter time to viral suppression in the denominator to estimate the ICER. We graphically present the variability in ICERs accounting for (1) the variability in estimated QALYs saved with a year of viral suppression, incorporating the reported confidence interval (0.273, 0.393) [22], and (2) the variability in reduced time to ART initiation under Rapid Start (one week to one month). We used the 2022 GDP per capita (US$76,399) as the threshold for cost-effectiveness per the World Health Organization [23].

## Results

The seven sites included in this analysis represent a variety of settings across the United States, including urban, rural, and suburban areas, community, health department and hospital-based clinics (Table 1). These sites serve demographically diverse HIV clinic populations ranging from fewer than 100 patients up to well over 3000 and provide a mix of clinical and social services. All sites implemented Rapid Start between 2015 and 2019, and all but one increased the number of persons initiating ART after implementation of Rapid Start (Table 2). UAB Birmingham 1917 Clinic, a large and long established HIV care and treatment program, experienced a small decline in the number of persons initiating ART during the initial and sustained Rapid Start implementation years assessed in this study. Notably, two sites, Kern County Health Officers Clinic and The Max Clinic, did not provide ART initiation services prior to implementation of Rapid Start.

**Table 2:** Number of persons who initiated ART and quality-adjusted life years (QALYs) saved by rapid start implementation period

	Number of persons initiating ART			QALYs saved, by contributing factor				Total QALYs saved*	
				Increased number initiating ART		Faster time to ART initiation*			
Site	Pre-	Initial	Sustained	Initial	Sustained	Initial	Sustained	Initial	Sustained
Asian Health Services	5	9	22	1.1	4.7	0.1, 0.3	0.1, 0.6	1.2, 1.4	4.9, 5.3
Borinquen Health Care	26	67	137	11.4	30.9	0.4, 1.9	0.9, 3.8	11.8, 13.3	31.8, 34.7
LGBT Life Center & CAN Community Health	18	30	58	3.3	11.1	0.2, 0.8	0.4, 1.6	3.5, 4.2	11.5, 12.7
Positive Care Center, Hennepin Healthcare	130	130	170	0.0	11.1	0.8, 3.6	1.1, 4.7	0.8, 3.6	12.2, 15.9
Kern County Health Officers Clinic	0	66	76	18.4	21.2	0.4, 1.8	0.5, 2.1	18.8, 20.2	21.6, 23.3
The Max Clinic	0	50	225	13.9	62.6	0.3, 1.4	1.4, 6.3	14.2, 15.3	64.1, 68.9
UAB Birmingham 1917 Clinic	129	102	111	−7.5	−5.0	0.7, 2.8	0.7, 3.1	−6.9, −4.7	−4.3, −1.9

* Ranges reflect assumed reductions in time between identification of HIV infection to initiation of ART with rapid start, from one week to one month

### QALYs Saved

The number of QALYs saved due to Rapid Start was largely attributable to increases in numbers of persons initiating ART at each site (Table 2). For example, five people initiated ART at Asian Health Services in the pre-implementation year, nine initiated during the initial year of implementation and 22 initiated during sustained Rapid Start implementation. During sustained implementation, this increase in numbers led to an additional 4.7 QALYs saved, whereas the QALYs saved due to reduced time to ART initiation alone ranged from 0.1 (for one week in reduced time) to 0.6 (for one month in reduced time). At the other end of the spectrum, The Max Clinic saved 62.6 QALYs in the sustained year by reaching 225 persons compared to 0 in the pre-implementation year, while QALYs saved due to the program providing Rapid Start compared to traditionally slower ART initiation ranged from 1.4 to 6.3; these factors together summed to an estimated 64.1 to 68.9 QALYs saved at The Max Clinic.

### Overall and Per-person Costs

Planning and implementation costs of Rapid Start varied across sites and over time (Table 3). In the initial year of implementation, per-person costs ranged from $646 at Positive Care Center, Hennepin Healthcare to $23,850 at The Max Clinic. During sustained implementation, Positive Care Center still had the lowest per-person costs ($595) while The Max Clinic benefitted from economies of scale and reduced per-person costs to $11,774, concurrent with an increase from 50 to 225 persons initiating ART. Asian Health Services had the highest per-person costs during sustained implementation ($12,736).

**Table 3:** Total costs, cost per-person and incremental cost per-person for planning, management, and provision of ART initiation services by rapid start implementation period

	Total cost of ART initiation by implementation period			Cost per-person of ART initiation by implementation period			Incremental cost per-person who initiated ART by implementation period	
	Pre-	Initial	Sustained	Pre-	Initial	Sustained	Initial	Sustained
Asian Health Services	$58,759	$119,249	$280,203	$11,752	$13,250	$12,736	$1,498	$985
Borinquen Health Care	$796,278	$625,945	$866,215	$30,626	$9,342	$6,323	($21,284)	($24,303)
LGBT Life Center & CAN Community Health	$146,955	$128,166	$128,352	$8,164	$4,272	$2,213	($3,892)	($5,951)
Positive Care Center, Hennepin Healthcare	$71,397	$84,032	$101,157	$549	$646	$595	$97	$46
Kern County Health officers clinic	$65,048	$137,845	$206,350		$2,089	$2,715	$2,089	$2,715
The Max Clinic	$29,594	$1,192,520	$2,649,041		$23,850	$11,774	$23,850	$11,774
UAB at Birmingham 1917 Clinic	$120,677	$173,880	$160,924	$935	$1,705	$1,450	$769	$514

Personnel effort was the primary contributor to costs across sites. Here we highlight programmatic features that impacted personnel costs for each site. Asian Health Services implemented Rapid Start with minimal pre-implementation planning. By the time of sustained implementation, medical service providers were able to efficiently provide same-day ART, while a growing support staff focused on linking clients to the program, providing wraparound services, and continued planning and management of Rapid Start. Borinquen Health Care Center provided significant case management and care coordination throughout all periods of study. During initial implementation of Rapid start, the program consolidated ART initiation services to one site with one medical provider. Within two years they returned to a distributed model of HIV care in five clinics with additional medical and mental health providers, further extending their reach.

LGBT Life Center & CAN Community Health devoted significant staff time to planning prior to implementation. During implementation, provider effort was consolidated into a single visit, while support staff time increased to facilitate services. Positive Care Center, Hennepin Healthcare was able to integrate Rapid Start into routine processes with minimal planning effort and minimal additional personnel effort across activities. Kern County Health Officers Clinic, which did not provide ART initiation prior to Rapid Start, devoted significant effort to pre-implementation planning. Due to the COVID-19 pandemic during initial implementation, providers spent minimal time with patients. The sustained year of implementation was characterized by longer visits and thus higher per-patient costs. The Max Clinic engaged in minimal devoted planning time pre-implementation, with many thoughtful discussions occurring “in the hallway”. The program rapidly scaled up services and staff (including clinicians, social workers, and health department specialists) during initial and sustained implementation. UAB Birmingham 1917 Clinic gradually implemented Rapid Start with no staff changes, learning and adapting the program over several years of implementation. In the initial year of Rapid Start, they devoted significant time to linkage to care; in subsequent years, they gradually shifted effort to follow-up care.

**Fig. 1:**
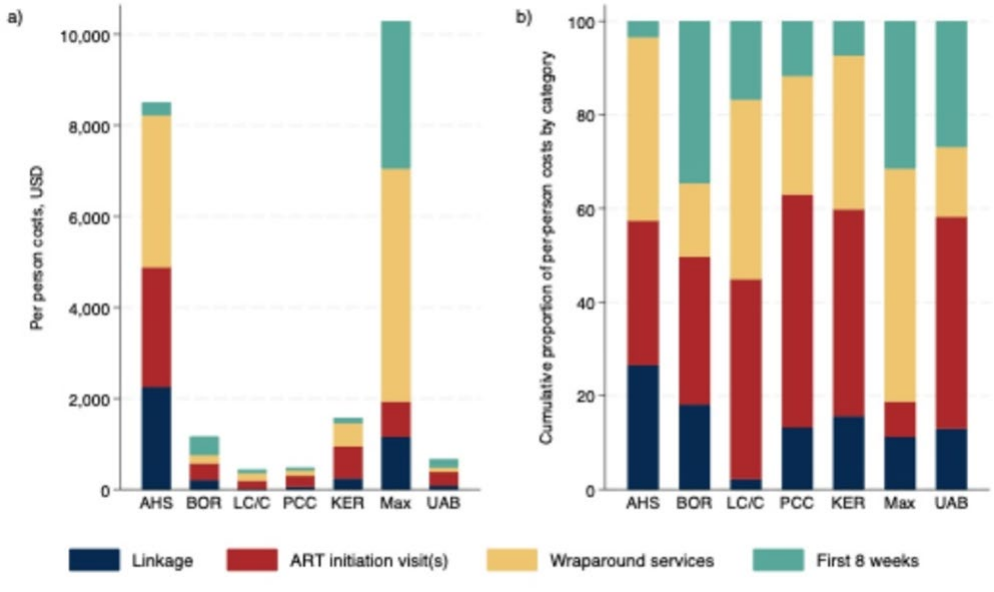
Site-level per-person costs during sustained implementation of Rapid Start for personnel effort devoted to antiretroviral treatment (ART) initiation services categorized by linkage to care, treatment initiation visits, wraparound services, and the initial eight weeks of follow-up. Panel (**a**) shows absolute per-person costs for each category. Panel (**b**) shows the proportion of costs for each category. *LC/C=LGBT* life center & can, *PCC* Positive Care Center, Hennepin; *UAB* UAB at Birmingham 1917 Clinic, *BOR* Borinquen Health Care, *KER* Kern county health officers Clinic, *AHS* asian health services, *MAX* the Max Clinic

ART initiation services, over planning or management, represented the largest category of personnel effort across sites. The distribution of personnel costs for ART initiation services in the sustained year of implementation are shown by site in in Fig. 1, categorized by linkage to care, clinical visits for ART initiation, wraparound services (assessments of housing, transit, mental health, benefits eligibility; enrollment in benefits; referral to support services), and initial follow-up HIV care (first eight weeks). The two programs with the highest per-person costs, Asian Health Services and The Max Clinic, had considerable costs attributed to the provision of wraparound services, while their clinical visits for ART initiation represented a relatively small fraction of per-person costs. Although Positive Care Center at Hennepin also devoted a relatively large proportion of effort to wraparound services, the absolute costs of these services were among the smallest.

### Incremental Cost Per-person

The largest relative change in per-person costs was observed at Borinquen Health Care Center, where per-person costs declined from $30,626 to $6,323 between pre- and sustained implementation, resulting in a cost savings of $24,303 for each person initiating ART under Rapid Start (Table 3). Rapid Start was also cost-saving at LGBT Life Center & CAN Community Health during sustained implementation (-$5,951 incremental per-person costs). Both sites increased the number of persons initiating ART by more than 3-fold and thus benefitted from economies of scale. Similarly, The Max Clinic significantly increased the number of persons served over time, however, incremental per-person costs were still high during sustained implementation due to not providing ART initiation before Rapid Start.

### Incremental Cost-Effectiveness

**Fig. 2:**
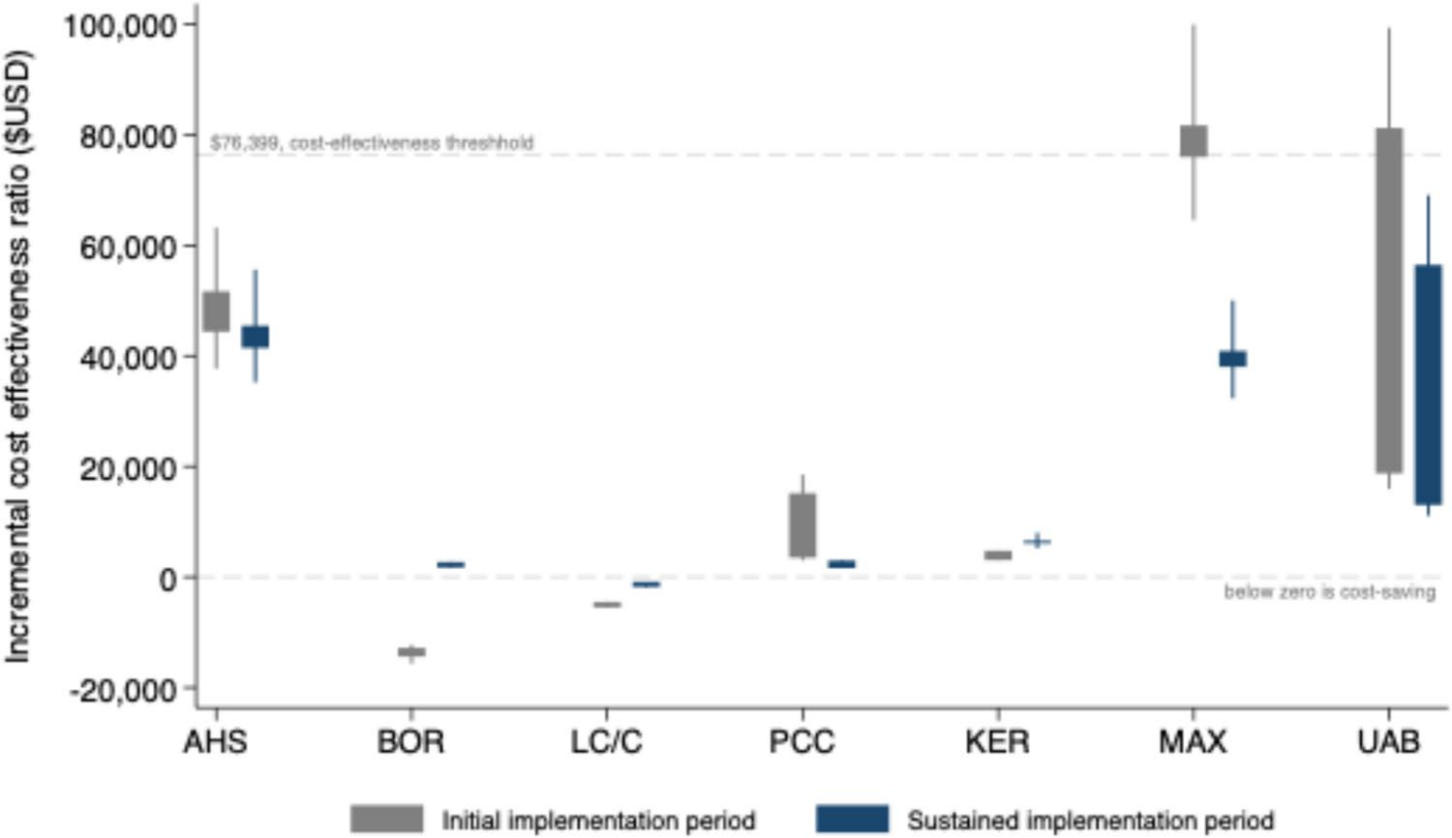
Incremental cost per QALYs saved by Rapid Start implementation year. Boxes represent the range of ICERs assuming viral suppression is achieved one week to one month faster under Rapid Start, and viral suppression saves 0.334 QALYs/year; Bars include variance from the confidence interval for the estimated 0.334 QALYs saved per year (0.273, 0.393). For UAB, these estimates are solely based on QALYs saved due to increased time virally suppressed. For all other sites, these estimates also include QALYs saved due to increased numbers of persons reached.* LC/C *LGBT life center & CAN, *PCC* *positive care cente*r, Hennepin, *UAB* UAB at Birmingham 1917 Clinic*,* *BOR* *borinquen health care,* *KER* Kern county health officers clinic, *AHS* Asian health services, *MAX* The Max Clinic

Rapid Start was cost-effective at five of seven sites during initial implementation (Fig. 2). The Max Clinic ($64,562 to $99,929 per QALY saved) was not cost-effective. At UAB Birmingham 1917 Clinic, because of declines in the number of persons initiating ART, cost-effectiveness was only estimated based on QALYs saved due to faster times to virologic suppression, resulting in considerable uncertainty in estimates. Thus in the initial year, estimated costs per QALYs saved ranged from $15,926 to $99,352, suggesting possible cost-effectiveness. By the time of sustained implementation, Rapid Start was cost-effective or cost-saving at all sites. During sustained implementation, UAB Birmingham 1917 Clinic was the most expensive site with an estimated $11,071 to $69,063 per QALY saved. LGBT Life Center & CAN Community Health was the most cost effective, with an estimated $1,374 to $1,785 cost savings per QALY saved.

## Discussion

Among seven diverse Ryan White funded sites throughout the United States, we found that Rapid Start was a cost-effective intervention for people with HIV. We estimated that implementation of Rapid Start was cost-effective (less than per capita GDP) or cost saving in five of seven programs during the initial year of implementation, and at all seven sites during sustained implementation. Rapid Start costs per patient were higher within sites providing significant wraparound services. while Rapid Start was more effective within programs that had larger increases in the number of people who initiated ART after implementation of Rapid Start.

In six out of the seven Rapid Start programs we observed, there were substantial increases in the number of persons who initiated ART over multiple years of implementation. To our knowledge, no other studies have examined whether Rapid Start leads to expanded uptake of ART. We speculate that Rapid Start removed barriers to initiation that some individuals face when return visits are required to initiate ART. Indeed, other public health interventions have observed increases in the number of persons receiving care when access is expanded or simplified. For example, in a randomized trial in Malawi, 51% of patients offered HIV self-testing in health facilities accepted same-day testing while waiting for other health services, compared to 14% of those offered one-on-one testing with a provider in control clinics [24]. Similarly, the Contraceptive CHOICE Project provided no-cost reversible contraception for 2–3 years in the St. Louis, Missouri area [25]. Clinics not only observed fewer unintended pregnancies and reduced rates of abortion among women who chose long-acting reversible contraceptive (LARC) options, but they also saw increases in LARC uptake with high continuation at 12 months (86%) and 24 months (77%).

The increase in the number of people who initiated ART was the largest contributor to additional QALYs saved in our study. Two programs, The Max Clinic and Kern County Health Officers Clinic, established ART initiation services concurrently with Rapid Start and thus inherently reached new clients with the provision of a new service. Both sites benefitted from close links to a broader network of clinical services. By the time of the sustained implementation year, The Max Clinic reached the largest number of persons initiating ART among all programs in our analysis. Borinquen Health Care Center in Miami-Dade County also observed large increases in persons initiating ART in each study period. In the initial year, their consolidation of wrap-around services at a single location enabled the site to efficiently meet the needs of a larger number of new clients. In turn, subsequent distribution of Rapid Start services to multiple sites, coupled with additional medical and mental health providers, allowed for continued increases in the number of people initiating ART during sustained implementation. On the other end of the spectrum, UAB 1917 Clinic observed a modest decline in the number of persons initiating ART over time. We expect this decline reflects natural annual variability in this large established program, though may also indicate that barriers to ART initiation were not significantly reduced at this site. Across sites, in contrast to the impact of increasing the number of people who initiated ART, our assumption that Rapid Start would lead to shorter times to viral suppression contributed to very few additional QALYs.

Per-person costs were partly driven by program stability and changes in complexity associated with implementation of Rapid Start programs. For example, both Positive Care Center at Hennepin Healthcare and UAB Birmingham 1917 Clinic were large, established providers of ART, with over 100 persons living with HIV initiating treatment in the pre-implementation year and over 2000 patients in care overall. Positive Care Center integrated Rapid Start into ongoing services with minimal additional staffing, while UAB 1917 slowly scaled up Rapid Start with no additional staffing. These two sites had the lowest per-person costs prior to implementation of Rapid Start as well as during the implementation years. At the other end of the spectrum, Asian Health Services, Borinquen Health Care Center and The Max Clinic had complex programs with significant wraparound services for vulnerable populations, resulting in high per-person costs.

All sites met the cost-effectiveness threshold proposed by the World Health Organization by the time of sustained implementation. While per-person costs are central to deriving cost-effectiveness, it is the additional, or incremental, costs of Rapid Start, coupled with QALYs saved, that drive the differences in cost-effectiveness estimates. Two sites, Positive Care Center at Hennepin and Kern County Health Officers Clinic fell well below this threshold, costing less than $20,000 per QALY saved. Positive Care Center reduced overall and per-person costs during Rapid Start. Thus, despite a stable number initiating ART in the first year of implementation and therefore a modest increase in QALYs saved, their program was cost highly effective. Kern County, on the other hand, which only incurred Rapid Start planning expenses in the pre-implementation year, more than doubled overall costs during implementation with modest per-person costs. However this site was among the highest in terms of QALYs saved due to the large increase in number of patients. At another two sites, Borinquen Healthcare and LGBT Life Center & CAN, Rapid Start was cost saving, where costs were either stable or declining while the number of persons served significantly increased. On the other hand, although AHS roughly doubled the number of persons initiating ART during each implementation year, though in absolute terms these increases were modest. As a small, relatively new, and growing program, they did not experience enough growth to reduce per-person costs over time, and therefore were modestly cost-effective by the sustained year of implementation. The Max Clinic, which experienced enormous growth, remained an expensive program with high per-person costs during sustained implementation. Programs that aim to reach the most disenfranchised, including persons who previously disengaged from HIV care, require significant investment in support services and will inherently have higher per-person costs. Finally, although UAB Birmingham 1917 Clinic had among the lowest incremental per-person costs, the numbers initiating ART modestly declined during implementation, resulting in minimal QALYs saved, which were estimated only based on assumed reduction in time to virologic suppression for the cost-effectiveness estimates.

This study has some limitations. First, we collected cost estimates after implementation of Rapid Start programs, thus costs associated with planning and early implementation are subject to recall bias. However, we asked sites to estimate costs using the same timeframe relative to the start of implementation of Rapid Start (e.g. July to June) each year, thus we anticipate that results reflect proportional changes in costs over time. Second, this study included sites that had been successfully providing Rapid Start services for at least two years. These results may not be fully representative of all sites that have attempted to implement Rapid Start. Nonetheless, the diversity of the sites included in this study lends itself to providing an overview of the variability in types of costs associated with Rapid Start implementation. Third, all sites included in the study are RWHAP-funded, which may reduce generalizability of these results to non-RWHAP-funded sites. Notably, our consistent finding of either cost-effectiveness or cost-savings in sustained implementation across a variety of clinical settings and regions in the United States is reassuring. Finally, for some sites, initial or sustained implementation occurred during the COVID-19 pandemic, which may have increased or lowered costs, such as with the use of virtual clinic appointments.

In summary, we found that Rapid Start programs in our study were either cost-effective or cost-saving once implementation of programs was established and sustained. These findings have significant implications for HIV care providers and policymakers, adding to the limited existing literature that emphasizes the cost-effectiveness of Rapid Start provision [15, 17, 26]. Importantly, the paper illustrates how, with minimal additional costs, QALYs were saved among patients at the seven sites, primarily attributed to increased ART initiation and a shortened time to viral suppression. With proven effectiveness in improving U.S. HIV outcomes through widespread and timely access to effective treatment, Rapid Start provision is now demonstrated as cost-effective in a variety of settings, making it an even more promising approach to impact the elimination of HIV.
